# “Trigger the Mind, Target the Gold”: Development and Validation of an ACPT (Acceptance and Commitment Performance Training) for Elite Shooters

**DOI:** 10.3390/bs16010052

**Published:** 2025-12-27

**Authors:** Suyoung Hwang, Woori Han, Eun-Surk Yi

**Affiliations:** 1 Department of Exercise Rehabilitation & Welfare, Gachon University, Incheon 21936, Republic of Korea; harriett0059@gmail.com; 2 Sport Science Center, Sports Town-gil 80, Chuncheon-si 24239, Republic of Korea

**Keywords:** ACPT-S, Psychological Skills Training (PST), Mindfulness-Acceptance-Commitment (MAC) approach, mindfulness, Fascial Circulation Exercise (FCE), Content Validity Ratio (CVR), psychological flexibility, elite shooters, applied sport psychology

## Abstract

Acceptance and Commitment Therapy (ACT) has been widely applied in clinical contexts; however, its systematic adaptation to elite sports, particularly precision-based disciplines such as shooting, remains underexplored. The present study aimed to develop and preliminarily validate an ACT-based psychological training program—the Acceptance and Commitment Performance Training for Shooters (ACPT-S)—by reframing ACT from a therapeutic intervention into a performance-oriented training framework. Using a multiphase formative evaluation design, a needs assessment was first conducted with 28 elite and collegiate shooters to identify sport-specific psychological demands. Based on these findings, a ten-session ACPT-S program was developed by integrating the six core ACT processes with shooter-specific routines, embodied exercises, and performance-relevant metaphors. The program was subsequently examined through two pilot studies: Phase 1 with four collegiate/corporate athletes and Phase 2 with 15 national-level shooters. Data were collected via session reflections, focus group interviews, and expert panel evaluations, and the Content Validity Ratio (CVR) analysis was used to assess conceptual clarity and implementation feasibility. The results indicated that ACPT-S was perceived as both feasible and contextually appropriate, with athletes reporting improvements in attentional focus, emotional acceptance, value-based motivation, and reduced anxiety. Qualitative analyses demonstrated strong engagement with ACT principles and their functional integration into shooting performance contexts, while all program components achieved CVR scores of ≥0.80, indicating a strong expert consensus. Program refinements were guided by feedback related to activity sequencing, metaphor resonance and personalization strategies. Overall, this study reconceptualizes ACT as a performance-enhancement framework rather than a purely clinical approach and introduces the ACPT-S as a novel, theory-driven, and scalable psychological training model for precision sports, providing a robust foundation for future longitudinal and comparative research.

## 1. Introduction

Shooting is an exceptionally demanding precision sport in which a deviation of only 1 mm at the muzzle can translate into several centimeters of error on the target, directly altering performance outcomes (Ihalainen et al., 2016). Beyond its technical requirements, shooting inherently contains unavoidable variability and error, as micro-fluctuations in tremor, respiratory rhythm, and environmental conditions are immediately reflected in performance outcomes. As a result, athletes repeatedly perform in high-pressure conditions where even minimal instability can determine competitive success or failure. From early developmental stages, shooters are evaluated almost exclusively on results, cultivating perfectionism, rigid performance tendencies, and heightened vulnerability to self-criticism (Hwang & Chang, 2022). These characteristics make shooting uniquely dependent on advanced psychological regulation—particularly sustained attentional control, emotional flexibility, and the capacity to cope constructively with evaluative pressure.

Importantly, this reliance on psychological regulation is not merely experiential but also neurocognitively substantiated. Recent findings show that elite shooters and archers exhibit enhanced efficiency in attention-related neural networks, suggesting that high-level performance in precision sports depends heavily on the stability and adaptability of attentional systems (Lu et al., 2021). Collectively, this evidence indicates that the psychological ecology of shooting requires training systems that go beyond technical routines to cultivate adaptive cognitive–emotional responses within a relentlessly evaluative environment.

Traditional Psychological Skills Training (PST) has been widely used to enhance performance across sports and commonly targets cognitive strategies such as imagery, self-talk, goal setting, biofeedback, and pre-performance routines. In shooting, PST interventions have been particularly evident in cue-based pre-shot routines, attentional focusing techniques, and structured breathing exercises designed to stabilize motor execution (Blumenstein & Orbach, 2015; Lee et al., 2023). While such methods provide tangible benefits, PST is rooted primarily in control and suppression models that aim to regulate arousal or eliminate unwanted thoughts and emotions. However, in static and high-precision contexts such as shooting, excessive attempts to control internal states may paradoxically narrow attentional bandwidth, destabilize fine motor coordination, and undermine trust in established routines. This critique aligns with Wegner’s (1994) theory of ironic mental control, which demonstrates that deliberate attempts to suppress internal experiences can increase their cognitive salience—an especially detrimental mechanism in precision sports where attentional misallocation can disrupt micro-motor execution.

These limitations have motivated the rise in acceptance-based approaches, which aim to foster more adaptive responses to internal experiences. Acceptance and Commitment Therapy (ACT), a third-wave behavioral approach grounded in Contextual Behavioral Science (CBS) and Relational Frame Theory (RFT), conceptualizes internal events not as obstacles to be removed but as behavioral phenomena to be acknowledged and integrated. ACT emphasizes six interrelated processes—acceptance, cognitive defusion, present-moment contact, self-as-context, values clarification, and committed action—which collectively constitute Psychological Flexibility (PF), defined as the capacity to persist or adapt behavior in the service of values despite difficult thoughts or emotions (Hayes, 2019). PF is distinct from mindfulness; whereas mindfulness involves present-moment awareness, PF represents a behavioral repertoire that enables athletes to respond effectively to contextual demands.

These theoretical advances directly informed the development of sport-specific models. Gardner and Moore (2004, 2007) introduced the Mindfulness–Acceptance–Commitment (MAC) approach as an adaptation of ACT for performance settings, providing an alternative to PST’s emphasis on suppression and control. Empirical studies have since demonstrated that ACT- and MAC-based interventions improve dispositional mindfulness, emotional regulation, and perceived performance across athletic contexts (Josefsson et al., 2019, 2020), with high-performance trials in ice hockey showing gains in PF, performance consistency, and resilience (Lundgren et al., 2020, 2021). Similar benefits have been reported in elite beach soccer (Sabzevari et al., 2023) and among adolescent high-performance athletes (Su et al., 2024). Systematic reviews further confirm that acceptance-based interventions uniquely contribute to resilience, adaptability, and performance enhancement (Noetel et al., 2019). Experimental evidence also supports the role of acceptance- and mindfulness-related processes in precision motor skill learning: in a randomized controlled trial, Zhang et al. (2016) found that mindfulness training enhanced fine-motor accuracy in dart throwing, a task structurally analogous to shooting.

Recent meta-analytic evidence strengthens the empirical foundation for ACT- and MAC-based interventions. Ptáček et al. (2024) reported significant improvements in PF, attentional stability, and competitive performance across athlete populations, while Wang et al. (2023) highlighted that mindfulness-based programs enhance athletic performance and emotional regulation, yet differ conceptually from ACT’s contextual behavioral foundations. Despite this progress, existing ACT and MAC programs remain limited in their translational depth: many lack embodied, repetitive structures; few integrate ACT processes directly into sport-specific motor routines; and most underemphasize values as transcendent reinforcers that motivate athletes to engage willingly with difficulty.

To address these limitations, the present study introduces the Acceptance and Commitment Performance Training (ACPT) model and its shooter-specific counterpart, ACPT-S. ACPT reorganizes ACT principles into a repetitive, movement-integrated system designed to align PF with the psychophysiological rhythms of elite shooting. Each ACT process is embedded within core components of shooting performance—acceptance through exposure to error and evaluative pressure, cognitive defusion through distancing from perfectionistic or self-critical thoughts, present-moment contact through respiratory phases and sighting drills, self-as-context through perspective-taking during pressure simulations, values clarification through personally meaningful performance standards, and committed action through replicable pre-shot and in-shot routines. By situating psychological processes within the fine-grained microstructure of shooting, ACPT-S advances ACT from a therapeutic paradigm toward a discipline-specific performance system.

These principles are further strengthened by the Fascial Circulation Exercise (ACPT-FCE) component, which supports neuromyofascial flow, reduces co-contraction, and enhances proprioceptive clarity. Prior applications in hybrid health models such as HOPE-FIT (Hwang & Yi, 2025) and Movement Poomasi “Wello!” (Hwang et al., 2025) suggest that such embodied practices enhance somatic–cognitive integration and rhythmic stability. Within this framework, PF serves as the mechanistic bridge linking ACT processes to performance. PF is conceptualized as a behavioral capacity through which athletes monitor internal cues, flexibly shift attention when disrupted, and re-anchor onto task-relevant stimuli. This monitor–shift–anchor cycle synchronizes with respiratory rhythms and the technical phases of shooting, allowing performers to accommodate internal variability while maintaining stability under pressure. This model aligns with recent emotion–technical integration frameworks in elite performance (Burns et al., 2019, 2022; Poulus et al., 2024), further supporting the relevance of acceptance-based training in precision sport contexts.

Accordingly, the present study has two primary aims: (1) to refine and evaluate the structural coherence and process validity of the ACPT-S framework, and (2) to examine expert evaluations of its theoretical integrity and sport-specific applicability. By situating ACPT-S within two decades of ACT/MAC research while addressing their translational limitations, this study advances ACT into a discipline-specific, movement-integrated performance system tailored for elite shooters.

## 2. Methods

### 2.1. Study Design

This study employed a multi-phase formative design to develop and preliminarily validate the Acceptance and Commitment Performance Training for Shooters (ACPT-S). The overall design was reconstructed and extended from Han’s doctoral dissertation (Han, 2022) and progressed through two sequential phases. Phase 1 focused on developing a sport-specific psychological training module and implementing an initial pilot application with elite and collegiate shooters. Phase 2 involved a structured expert evaluation in which ACT specialists, sport psychologists, and national-level shooting coaches assessed the theoretical coherence, procedural structure, and practical feasibility of the module.

The study followed a formative logic model in which each stage informed the next through a cyclical process of needs assessment, theoretical alignment, module construction, iterative refinement, pilot implementation, and subsequent program adjustment. This design was purposefully selected to integrate the contextual demands of precision shooting with Acceptance and Commitment Training principles, thereby ensuring both ecological validity and theoretical rigor. The application of ACT/MAC principles to shooting was further justified by the sport’s distinct psychophysiological characteristics: elite shooting requires the fine-grained synchronization of respiratory rhythms, attentional stability, and micro-motor precision under sustained evaluative pressure. Although the MAC protocol has been successfully applied in various sporting contexts (Gardner & Moore, 2004, 2007; Josefsson et al., 2019, 2020, 2024; Lundgren et al., 2020, 2021), ACPT-S extends this framework by embedding ACT’s six core processes directly into shooter-specific, embodied routines such as breath–release cycles, attentional anchoring drills, and trigger-alignment metaphors. Within this program, Psychological Flexibility (PF) was operationalized not as an abstract construct but as a metacognitive attentional mechanism that supports adaptive performance behaviors.

**Figure 1:**
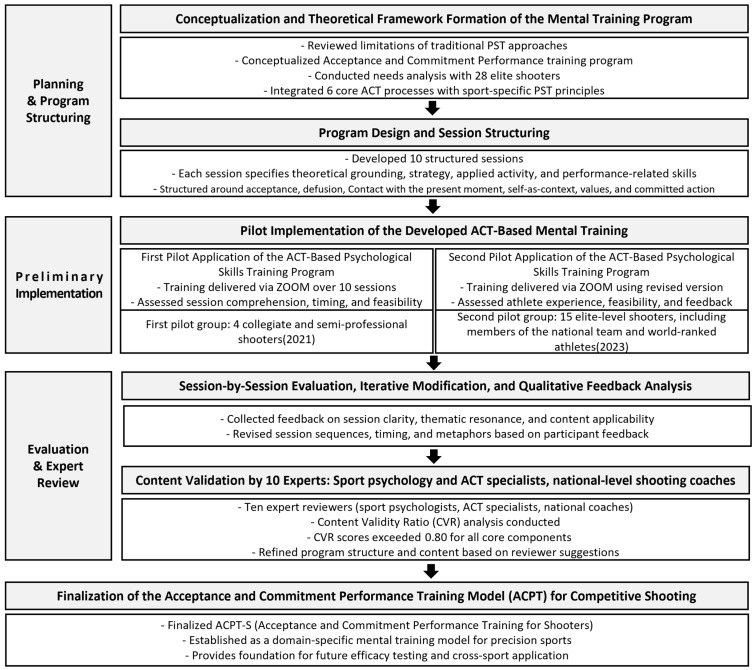
Overall training development and implementation process.

Figure 1 visualizes the formative, multi-phase structure of ACPT-S development, illustrating the progression from conceptualization to program refinement and expert validation. In contrast, Table 1 summarizes the experimental structure (Q_1_–X_1_–Q_2_) applied specifically in the pilot component of the design, capturing the pre-test, intervention, and post-test sequence used in both phases. Together, these components clarify the methodological organization of the study and delineate the distinct roles of developmental and evaluative procedures within the overall research framework.

### 2.2. Participants and Recruitment

#### 2.2.1. Needs Assessment Participants

To identify discipline-specific psychological demands and inform the initial construction of the ACPT-S framework, a total of 28 elite and collegiate-level shooters (rifle and pistol disciplines) voluntarily completed a semi-structured written survey. The survey examined (a) psychological challenges encountered during training and competition, (b) prior exposure to psychological skills training, and (c) expectations regarding the structure and content of a shooting-specific psychological program.

Open-ended responses were thematically analyzed to identify recurrent psychological needs, including perfectionism, attentional lapses under pressure, and difficulties accepting internal experiences. These themes directly informed the development of ACPT-S session goals, the selection of core ACT processes, and the construction of sport-specific metaphors incorporated into the training protocol.

#### 2.2.2. Pilot Study Participants

The ACPT-S pilot testing was conducted in two sequential phases, each serving distinct formative purposes within the overall development process.

- Phase 1 (2021)
  This initial phase aimed to examine the structural coherence and practical feasibility of the preliminary ACPT-S draft. The study was approved by the Korea National Sport University IRB (No. 1263-202106-HR-072-01). Four athletes (2 male, 2 female) from collegiate and corporate shooting teams participated in ten online training sessions delivered via Zoom. Participants’ mean age was 23.0 years, with an average of 9.0 years of competitive experience. Detailed demographic information is presented in Table 2.
- Phase 2 (2023–2024)
  The second phase focused on evaluating the refined ACPT-S program with high-performance athletes exposed to elite international competition demands. This phase was approved by the Hanshin University IRB (No. 2023-02-006). Fifteen national-level shooters—including several athletes ranked within the global top tier—completed ten online sessions. The sample consisted of pistol shooters (4 male, 3 female) and rifle shooters (4 male, 4 female). Participants’ mean age was 29.4 years, and their average competitive experience was 15.5 years. Participant characteristics are summarized in Table 3.

### 2.3. Intervention Overview

The Acceptance and Commitment Performance Training for Shooters (ACPT-S) was developed as a domain-specific adaptation of Acceptance and Commitment Training (ACT), in which the six core processes were systematically translated into the perceptual–motor, attentional, and emotional demands of precision shooting. Rather than applying ACT as a generic psychological framework, ACPT-S embeds each process into shooter-specific routines—such as breath–trigger synchronization, stability under micro-tremor, and tolerance of internal fluctuations during aiming—thereby extending ACT principles into an embodied, performance-relevant context consistent with functional contextualist perspectives.

To ensure discipline-specific precision and functional contextual alignment, the intervention was grounded in three tailoring principles.

First, embodied implementation linked ACT processes with brief sensory–motor grounding procedures—such as breathing–movement coupling, subtle postural micro-adjustments, proprioceptive noticing, and neuromyofascial steadiness—that enhance present-moment awareness during the aiming phase.

These procedures were not physical conditioning elements but brief perceptual–motor grounding tasks designed to facilitate ACT processes (e.g., present-moment contact, defusion) in situations where micro-instability is unavoidable.

Second, precision-task relevance ensured that psychological processes directly mapped onto core shooting actions, including pre-shot routines, sight-picture maintenance, trigger execution, and recovery following errors. Values clarification was incorporated into this mapping to help athletes align technical routines with personally meaningful performance intentions.

Third, contextual pressure simulation incorporated evaluative cues (e.g., countdowntiming, observer presence, performance-contingent feedback) to approximate competitive stressors and create opportunities for practicing acceptance, defusion, and committed action under realistic performance conditions.

Each session followed a structured five-component sequence that operationalized ACT within the shooting domain:

1. Conceptual grounding. Core ACT processes (e.g., acceptance, defusion, values clarification) were introduced using scenarios derived from aiming instability, error fixation, and perfectionistic control attempts commonly reported by shooters.
2. Experiential task. Athletes engaged in perceptual–motor exercises—such as breath–release cycling, proprioceptive anchoring, “sight-picture acceptance drills,” and “cognitive-noise labeling”—that linked ACT processes with the intrinsic sensory demands of precision tasks.
3. Metaphor integration. Shooter-specific metaphors (e.g., “fogged lens,” “holding water in open hands,” “recoil without resistance”) were used to promote functional contextual understanding and replace generic ACT metaphors with performance-relevant analogues.
4. Reflective consolidation. After each activity, athletes documented attentional shifts, emotional triggers, proprioceptive cues, and bodily sensations observed during simulated shots to deepen meta-awareness and integrate learning.
5. Performance linkage. ACT processes were functionally mapped onto shooting-relevant behaviors—including pre-shot stabilization routines, trigger control, tolerance of micro-instability, and recovery from performance errors—clarifying how psychological flexibility supports technical execution and consistent performance.

**Figure 2:**
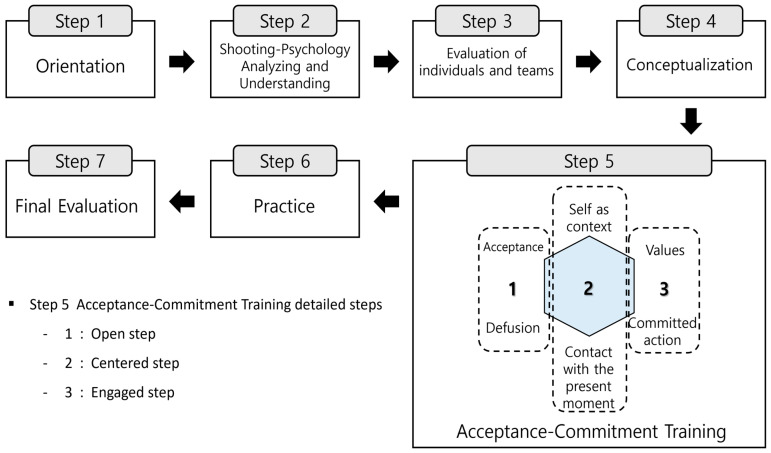
Final Theoretical Model of Acceptance and Commitment Performance Training Program.

**Figure 3:**
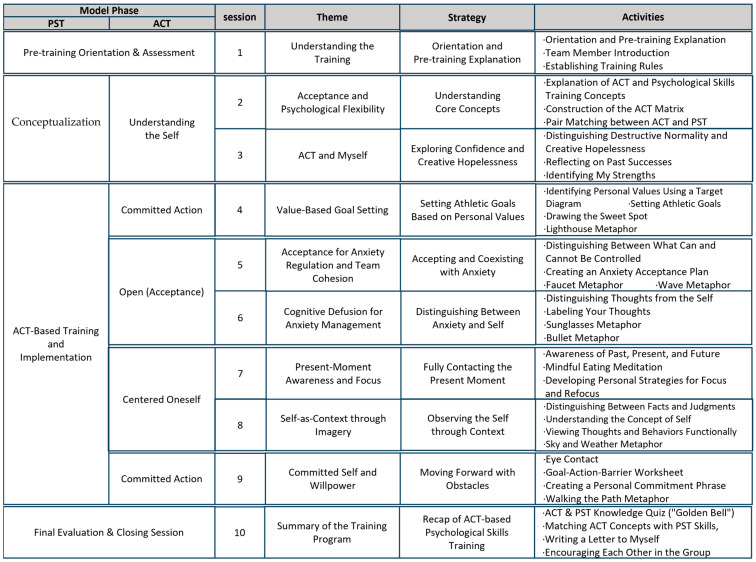
Session-by-Session Structure of the ACPT-S Program (10 Sessions).

Figure 2 provides a visual consolidation of how the six ACT processes, the three tailoring principles, and the embodied shooting routines interact within the ACPT-S framework. Figure 3 further translates this theoretical structure into a ten-session progression, showing how each component is operationalized across the program. These Figures serve as structural guides linking the conceptual model to its applied training sequence.

### 2.4. Procedures

#### 2.4.1. Needs Assessment Procedures

The needs assessment was conducted to identify discipline-specific psychological demands prior to the construction of the ACPT-S framework. All 28 participants completed two open-ended written questions:

(1) “What psychological aspects do you feel need improvement?”
(2) “What kind of support would you like to receive from psychological skills training?”

Responses were subjected to preliminary thematic categorization to extract performance-relevant needs, including perfectionism-driven self-pressure, attentional instability during high-evaluation moments, and difficulties in emotional acceptance. These themes directly informed the refinement of ACT process emphasis, session objectives, and sport-specific metaphors integrated into the ACPT-S protocol.

#### 2.4.2. Pilot Implementation Procedures

Phase 1 (2021)

The preliminary ACPT-S draft was delivered to four collegiate and corporate-team athletes through ten 60 min online sessions. Sessions were delivered via Zoom under IRB approval (Korea National Sport University, No. 1263-202106-HR-072-01).

After each session, athletes completed written feedback evaluating comprehension, clarity, metaphor fit, and perceived applicability to shooting tasks. These reflections informed iterative modifications to session sequence, pacing, and grounding activities. Participant characteristics are summarized in Table 2.

Phase 2 (2023–2024)

The refined ACPT-S protocol was implemented with fifteen national-level athletes, including internationally ranked shooters, under IRB approval (Hanshin University, No. 2023-02-006). Ten 60 min online sessions were delivered.

Following each session, participants completed reflective self-assessment forms rating attentional control, anxiety regulation, and session relevance on a 5-point Likert scale. Semi-structured interviews were administered to gather detailed experiential insights regarding how athletes applied ACT processes to perceptual–motor shooting tasks.

Upon program completion, a Focus Group Interview (FGI) was conducted to elicit integrative reflections on training engagement, perceived effectiveness, and contextual fit. Throughout both phases, the lead researcher documented observations in analytic field notes, focusing on emotional responses, attentional shifts, and changes in psychological skills awareness. Characteristics of Phase 2 participants are provided in Table 3.

### 2.5. Measures and Data Sources

Data collection incorporated quantitative, qualitative, and expert-evaluation sources:

- Self-report: Post-session questionnaires (5-point Likert scale) assessing attentional control, anxiety regulation, and perceived session relevance.
- Qualitative data: Open-ended survey responses (needs assessment), session reflections, semi-structured interviews, FGI transcripts, and researcher field notes
- Expert review: Training manuals, worksheets, and evaluation sheets reviewed by a panel of experts. Expert panel demographics are summarized in Table 4.

Interview protocols were constructed using a structured item-development framework to ensure depth, clarity, and alignment with qualitative research standards (Taylor, 2017).

### 2.6. Data Analysis

**Figure 4:**
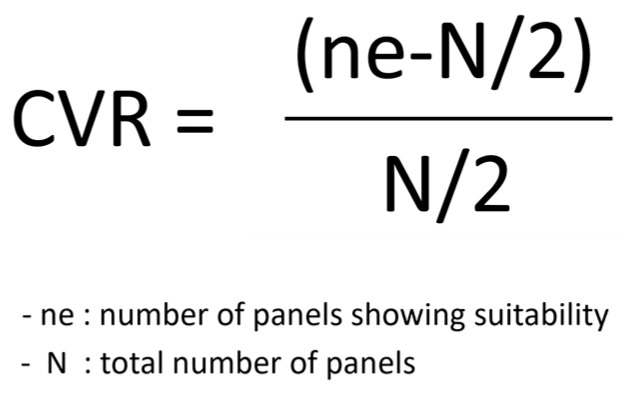
CVR Calculation Formula (Lawshe, 1975).

- Quantitative Analysis: Post-session questionnaire data were analyzed using descriptive statistics (means and standard deviations) to summarize athletes’ ratings of attentional control, anxiety regulation, and session relevance. No inferential statistics were conducted due to the exploratory and small-sample nature of the pilot design.
- Qualitative Analysis: All qualitative materials—including needs assessment responses, session reflections, semi-structured interviews, FGIs, and researcher field notes—were analyzed using qualitative content analysis (QCA) with an interpretive–reflexive approach (Schreier, 2012). This analytic strategy was selected because the concise, experience-focused nature of the data aligned more closely with the identification of meaning units and higher-order content categories, rather than the development of abstract themes required in reflexive thematic analysis (Braun & Clarke, 2006, 2022).
  Analysis proceeded through the following iterative steps: (1) repeated familiarization with all textual materials; (2) inductive coding of meaning units relevant to emotional, attentional, and performance-related processes; (3) grouping of codes into coherent categories that captured shifts in acceptance, attentional focus, cognitive flexibility, and psychological insight; (4) refinement of category boundaries through constant comparison; and (5) selection of representative quotations to illustrate each category.
  Throughout the analysis, a reflexive stance was maintained to acknowledge the researcher’s interpretive role, and analytic decisions were documented to ensure transparency. Method triangulation across multiple data sources enhanced credibility and supported convergence of findings. The qualitative findings are reported in the Results section.
- Content Validity: Lawshe’s (1975) Content Validity Ratio (CVR) was computed for expert panel ratings, using the established threshold of 0.62 for a 10-member review panel. The calculation formula is depicted in Figure 4.

### 2.7. Trustworthiness and Ethical Considerations

#### 2.7.1. Ethical Approval and Participant Rights

This study was conducted in accordance with the ethical standards of the Declaration of Helsinki and received approval from the Institutional Review Boards (IRBs) of Korea National Sport University (IRB No. 1263-202106-HR-072-01; Approval Date: 17 June 2021) and Hanshin University (IRB No. 2023-02-006; 13 November 2023). Phase 1 of data collection occurred from June 17 to 6 November 2021, and Phase 2 from 13 November 2023 to 17 June 2024.

All participants received detailed information regarding the study’s purpose, procedures, and voluntary nature. Informed consent was obtained prior to participation. Athletes voluntarily completed surveys, activity worksheets, and feedback forms, and their confidentiality and anonymity were strictly protected throughout the study. No identifying information was retained in the analysis or reporting process, and all data were securely stored in accordance with institutional policies.

#### 2.7.2. Trustworthiness and Rigor

To establish the trustworthiness of this qualitative inquiry, the study employed multiple validation strategies grounded in Guba and Lincoln’s (1994) four foundational criteria: credibility, transferability, dependability, and confirmability. The study was further guided by a subtle realist and experiential–contextual qualitative stance, reflecting an understanding that participants’ accounts provide situated yet meaningful access to their psychological experiences. Building upon these principles, we further integrated contemporary insights from Adler (2022), who emphasized that trustworthiness must be understood as a reflexive, dynamic, and ethically engaged process that is maintained throughout the lifecycle of the research.

To enhance credibility, data triangulation was performed using open-ended surveys, semi-structured interviews, session-specific reflective feedback, and observational field notes. This multi-perspective approach fostered analytic depth and internal coherence (Breitmayer et al., 1993; Donkoh & Mensah, 2023). Investigator triangulation via multi-analyst coding, cross-checking, and consensus meetings was used to minimize interpretive bias and ensure analytic robustness.

Member checking involved returning thematic summaries to a subset of participants to verify resonance, clarity, and authenticity (Birt et al., 2016). This process reinforced alignment between researcher interpretation and athlete experience. To promote transferability, the study provided detailed accounts of participant characteristics, training contexts, and procedural steps, enabling readers to assess applicability to similar elite sport settings.

Dependability was enhanced through maintaining an explicit audit trail documenting iterative program revisions, methodological decisions, and analytic procedures. Confirmability was supported by reflexive journals and external expert review, ensuring that interpretations remained grounded in the data rather than researcher assumptions. Feedback from ACT specialists and sport psychologists helped verify fidelity to ACT principles while securing cultural and sport-specific relevance (Hayes et al., 2011).

Overall, the study adhered to established standards of qualitative rigor while adopting modern perspectives that frame trustworthiness as an evolving, ethically situated practice (Adler, 2022). All procedures followed IRB approval and conformed to international ethical guidelines.

## 3. Researcher Positionality

This study was conducted by Dr. Suyoung Hwang and Dr. Woori Han, both Ph.D.-level scholars in sport psychology. They collaborated with a shared practitioner-scholar orientation, committed to promoting the psychological well-being and performance enhancement of elite athletes by bridging research and applied practice.

Dr. Woori Han initially conceptualized the Acceptance and Commitment Performance Training framework for elite shooters in her 2021 doctoral dissertation—the first of its kind in Korea. Her earlier work included designing ACT-based interventions to promote psychological flexibility in middle- and long-distance runners during her master’s program and developing an ACT training program to help college athletes manage COVID-19-related stress during her doctoral studies. Through years of field application and academic engagement, Dr. Han has cultivated top-tier expertise in ACT implementation within sport contexts.

She holds a Level 1 certification in sport psychology counseling (KSSP), has completed both the basic and advanced sport mental coaching curricula, and has provided individual and group consultations to elite athletes across track and field, judo, archery, badminton, and e-sports. In addition, she actively participates in these disciplines as a recreational athlete to deepen her experiential insight. A certified ACT Level 2 practitioner, Dr. Han has been actively involved in national psychology conferences and workshops since 2015 and has delivered over 100 ACT-based sessions through the Gangwon Sports Council, supporting performance enhancement beyond the shooting domain.

Dr. Suyoung Hwang is a former national skeet shooter with over a decade of elite-level experience. She holds an official shooting coach license and has completed the Level 1 sport psychology counseling curriculum through KSSP. As a research professor, she bridges academic inquiry with athletic practice. Dr. Hwang also holds an internationally accredited MBTI certification and actively engages in professional development across mindfulness, ACT, and performance-based psychological interventions. In this study, she contributed significantly to strengthening the structural design and validating the program through both qualitative and quantitative analyses.

As insider researchers, both authors possess a profound understanding of the psychological and cultural nuances within the Korean elite sport system. Aware of the potential for interpretive bias, they implemented multiple strategies—including triangulation, external consultation, and independent coding procedures—to ensure analytical rigor and uphold the trustworthiness of the research (Morrow, 2005).

## 4. Results

### 4.1. Sample Characteristics

Participants’ demographic and athletic profiles for both pilot phases are summarized in Table 2 (Phase 1, 2021) and Table 3 (Phase 2, 2023–2024).

Phase 1 consisted of four collegiate and corporate team shooters (2 male, 2 female; mean age = 23.0 years; mean competitive experience = 9.0 years). This phase primarily served to examine preliminary feasibility, structural coherence, and early usability of the ACPT-S draft.

Phase 2 included fifteen national-level athletes (7 pistol, 8 rifle)—several with international competition experience and top-tier world rankings (mean age = 29.4 years; mean competitive experience = 15.5 years). These participants represented a highly specialized population, providing an appropriate context to validate the refined intervention under elite performance demands.

Across both cohorts, the diversity of competitive background enabled a multi-layered validation, in which Phase 1 generated initial implementation insights and Phase 2 demonstrated applicability within a high-performance environment.

### 4.2. Needs Assessment Validation

Qualitative content analysis of the open-ended needs assessment responses (N = 28) generated several recurrent psychological categories reflecting the challenges faced by competitive shooters. The most frequently identified needs pertained to worry and rumination (21.4%), anxiety and tension (21.4%), and reduced mental toughness (21.4%), which emerged as dominant content categories capturing athletes’ cognitive and emotional strain during training and competition. Additional categories included difficulties in concentration, emotional regulation, and self-confidence, as summarized in Table 5.

When asked about preferred forms of psychological support, athletes most commonly emphasized the need for mental toughness enhancement (42.9%), followed by tension management (14.3%), anxiety regulation (14.3%), and concentration-related support (7.1%) (see Table 6). These patterns highlighted not only the psychological vulnerabilities embedded within elite precision sports but also athletes’ dissatisfaction with generic PST approaches and their desire for interventions that reflect individual differences and contextual specificity.

Collectively, these findings served as the empirical foundation for the development of the hybrid theoretical model (see Section 4.3). The categories derived from the needs assessment directly informed decisions regarding process emphasis, metaphor selection, and session sequencing within the ACPT-S framework, ensuring alignment with athlete-derived priorities.

### 4.3. Theoretical Model Output

Needs assessment results and prior literature were synthesized into a hybrid theoretical framework that integrates the six core processes of Acceptance and Commitment Training (acceptance, cognitive defusion, present-moment contact, self-as-context, values, and committed action) with established educational progressions from Psychological Skills Training (Morris & Thomas, 1995; Boutcher & Rotella, 1987).

The resulting ACPT-S model (Figure 2) provides a participant-centered and context-sensitive conceptual structure specifically aligned with the static, perceptual–motor, and precision-based demands of elite shooting. This framework was subsequently translated into a ten-session draft program (Figure 3), in which ACT processes and PST principles are operationalized into sequential experiential, reflective, and performance-linkage activities suitable for elite shooting contexts.

### 4.4. Pilot Validation

#### 4.4.1. Operational Evaluation

Across both phases, program implementation was feasible, and the session structure generally unfolded as intended. Several procedural issues emerged, including insufficient explanation of skill transfer during Session 3 and activity overload in Session 4, which contributed to time overruns. These findings indicated the need for clearer instructional scaffolding and more balanced activity sequencing.

Minor technical disruptions (e.g., occasional internet instability) did not impede overall delivery. Notably, athletes in Phase 2 demonstrated progressively higher engagement and concentration, supporting the practical viability of the online format even under competitive performance schedules.

#### 4.4.2. Process Evaluation (QCA)

Analysis of participants’ reflective writings and session feedback revealed several recurring categories of experiential benefit:

1. Emotional acceptance and cognitive perspective-taking—Participants described greater ease in observing internal events without judgment, contributing to reduced emotional reactivity.
2. Enhanced attentional control and self-awareness—Athletes reported increased clarity in noticing thought–emotion interactions during aiming-related tasks.
3. Strengthened self-trust and conceptual understanding—Defining ACT concepts in their own words was perceived as meaningful and personally grounding.
4. Positive engagement with value-based and individualized tasks—Small group formats facilitated deeper participation and relevance.

Illustrative quotations supporting each content category are presented in Table 7.

#### 4.4.3. Outcome Evaluation (QCA)

Post-program interviews and Focus Group Interviews yielded four overarching categories of psychological change:

1. Internalization of ACT principles—Participants reported improved cognitive defusion, reduced thought believability, and increased psychological flexibility.
2. Selective attentional focus—Athletes emphasized focusing on controllable elements while deliberately disengaging from unproductive concerns.
3. Tolerance of negative emotions—Reflections emphasized reduced rumination and greater willingness to experience emotional discomfort during performance situations.
4. Temporal self-awareness—Participants described improved integration of past experiences, present cues, and future intentions within their performance routines.

**Figure 5:**
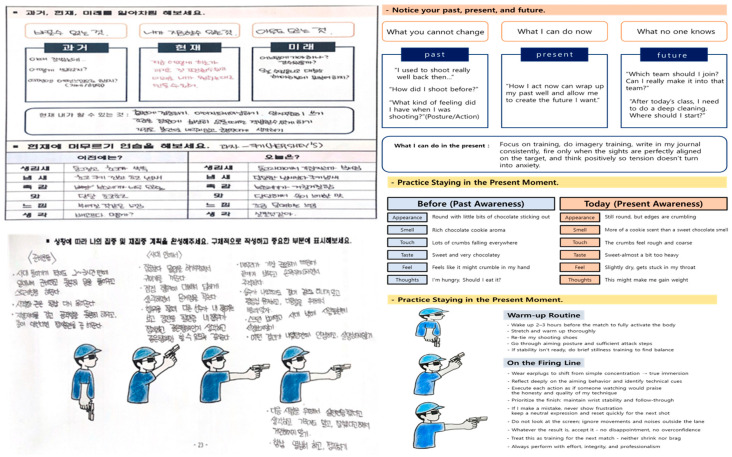
Representative example from the 10-session ACPT program. Note. Non-English (Korean) terminology is retained to preserve ecological and cultural validity, as all sessions were delivered in Korean. English descriptions are provided to facilitate comprehension.

Representative quotations are summarized in Table 8. A visual example of applied session activity (Session 8) is provided in Figure 5.

Collectively, these operational, process, and outcome evaluations justified modifications to activity load, refinement of metaphors, and strengthened reflective components—directly contributing to the iterative finalization of the ACPT-S protocol.

### 4.5. Expert Validation

A panel of ten experts—eight ACT-trained sport psychologists and two national-level shooting coaches—evaluated the refined ACPT-S draft to assess conceptual clarity, contextual appropriateness, and practical feasibility. Content Validity Ratio (CVR) scores exceeded the recommended threshold of 0.62 for a ten-member panel across all program items, with each item rated at CVR ≥ 0.80. These findings indicate a high level of expert consensus regarding the necessity and appropriateness of the intervention components (see Figure 4 and Table 9).

Qualitative feedback offered by the panel reinforced several core considerations for final refinement. Experts emphasized the value of more explicit explanation of ACT concepts to enhance theoretical accessibility, recommended strategic redistribution of activity time to prevent cognitive overload and maintain engagement, and highlighted the importance of deepening reflective elements rather than relying on a high volume of activities. They also encouraged the inclusion of tasks that athletes could continue practicing autonomously outside the sessions, to strengthen learning transfer into daily training routines. A consolidated synthesis of these recommendations is presented in Table 10.

Together, the CVR results and qualitative synthesis confirmed that the ACPT-S structure was conceptually sound, theoretically coherent, and aligned with the functional demands of precision shooting.

### 4.6. Program Finalization as Validation Output

**Figure 6:**
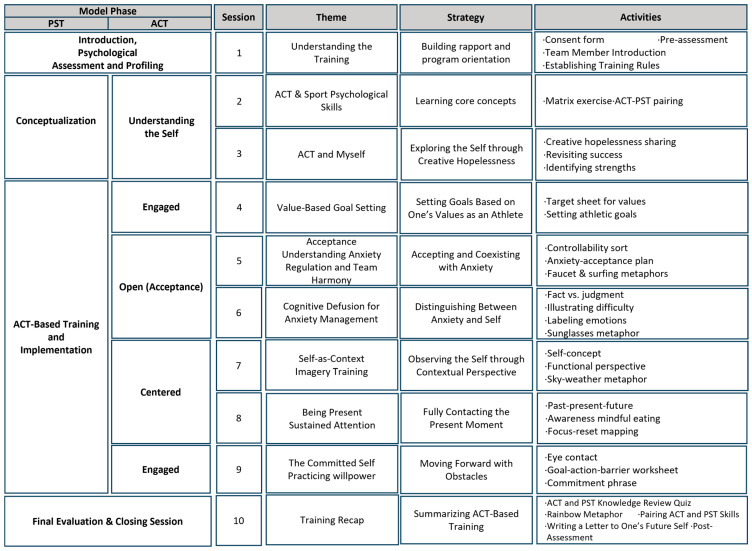
Final ACPT-S (Acceptance and Commitment Performance Training for Elite Shooters) Program.

Drawing upon iterative evidence from needs assessment, two-phase pilot testing, and expert evaluation, the ACPT-S protocol was finalized into a standardized ten-session model. Each session adhered to a consistent five-component structure comprising conceptual overview, experiential activity, metaphor application, individual reflection, and performance linkage. The finalized structure is depicted in Figure 6.

Importantly, finalization represented not merely the completion of a program draft but the culmination of a multi-layered formative validation process. Activity and metaphor selection were systematically grounded in ACT and PST literature (Johnson et al., 2023; Kang, 2016; Son, 2020; Stoddard & Afari, 2014) and were further refined through cultural adaptation to the lived experiences and performance routines of Korean elite shooters. Pilot findings provided granular insight into the feasibility and functionality of the activities—such as the intuitive utility of “Creative Hopelessness Sharing,” the clarity afforded by “Fact vs. Judgment,” and the sport-specific resonance of the “Sky and Weather Metaphor.”

Expert evaluation subsequently affirmed the conceptual fidelity of these components to ACT principles while underscoring the importance of clearer time allocation and stronger integration with daily training. These insights were reflected in the final session sequencing and instructional scaffolding.

Thus, program finalization marks the closure of a comprehensive validation cycle that integrated multiple data sources: needs assessment results (Table 5 and Table 6), iterative pilot testing with feedback loops (Table 2, Table 3, Table 7 and Table 8), and expert CVR analysis with qualitative synthesis (Figure 4, Table 9 and Table 10). Through this cumulative evidence base, the ACPT-S was validated as both theoretically robust and practically feasible, providing a high-fidelity intervention ready for subsequent large-scale empirical testing.

### 4.7. Session-Level Program Highlihts

The validated ACPT-S framework was translated into session-level activities that embedded ACT processes within the perceptual–motor microstructure of shooting performance. Each thematic component represented a theoretically grounded and empirically supported element refined through pilot implementation and expert evaluation.

Self-understanding was cultivated through activities such as Creative Hopelessness Sharing and the ACT Matrix (Krafft et al., 2017), which prompted athletes to examine the functional limits of avoidance-based coping and to articulate internal barriers to performance. The introduction of self-compassion further strengthened emotional resilience, consistent with evidence that self-compassion enhances confidence and regulates anxiety in elite performance settings (Terry & Leary, 2011).

Values clarification was introduced early through the Value Target Tool, enabling athletes to link personal meaning with performance intentions. This design aligns with recent findings that value-driven goal structures reinforce intrinsic motivation and sustain effort under evaluative pressure (Su et al., 2024). By mapping values to concrete training behaviors, shooters reported increased clarity and persistence during high-stress tasks.

Acceptance processes were trained using metaphors such as the faucet and the Olympic surfing wave, combined with individualized Anxiety–Acceptance Plans. These strategies fostered openness toward internal fluctuations during aiming, paralleling evidence that acceptance promotes cognitive flexibility and reduces maladaptive rumination (Sabzevari et al., 2023).

Cognitive defusion was strengthened through exercises such as Fact vs. Judgment and the Sunglasses Metaphor, which facilitated flexible distancing from intrusive or anxiety-laden thoughts. This approach echoes prior findings showing that defusion reduces thought believability and enhances attentional allocation to task-relevant cues—mechanisms particularly essential in precision sports (Shulze, 2017; Stoddard & Afari, 2014).

Present-moment awareness was embedded through activities such as Noticing Past–Present–Future and mindful sensory tasks (e.g., eating meditation). These exercises supported attentional anchoring, reflecting evidence that present-focused awareness enhances attentional network efficiency in shooters and archers (Lu et al., 2021).

Self-as-context was developed using the Sky and Weather Metaphor, guided imagery, and functional perspective-taking tasks. Athletes reported expanded metacognitive awareness and greater tolerance of fluctuating emotional states, consistent with research indicating that decentered awareness promotes psychological stability in high-performance contexts (Tóth et al., 2024).

Committed action was reinforced through Goal–Action–Barrier planning and commitment phrase work, which operationalized the transition from abstract values to specific, repeatable training behaviors. This process ensured alignment between values, technical routines, and performance execution, mirroring recent ACT-based sport interventions that highlight the central role of committed action in sustaining behavioral persistence (Pears & Sutton, 2021).

Collectively, these session-level components functioned not as descriptive activities but as validated intervention mechanisms, consistently corroborated through pilot feedback (Table 7 and Table 8) and expert review (Table 9 and Table 10). In contrast to conventional PST approaches, which emphasize control, suppression, or symptom reduction, ACPT-S integrated acceptance-based psychological flexibility processes into the embodied routines of precision shooting—bridging metacognitive flexibility with neuromotor steadiness and the attentional demands of elite performance.

## 5. Discussion

The present study developed and preliminarily validated the Acceptance and Commitment Performance Training for Shooters (ACPT-S), a domain-specific psychological skills program grounded in Acceptance and Commitment Training (ACT) and informed by the performance demands of precision shooting. Whereas ACT has been primarily applied in clinical settings, the ACPT-S extends ACT into a structured performance-training model that targets psychological flexibility, attentional stability, and value-driven motivation within high-performance sport environments. This reconceptualization—transitioning from therapeutic intervention to performance enhancement—represents a substantive theoretical contribution to the emerging literature on acceptance-based training in elite sport.

In contrast to traditional Psychological Skills Training (PST), which focuses on cognitive control strategies such as goal-setting, arousal regulation, and attentional routines, the ACPT-S incorporates acceptance-based mechanisms that enable athletes to respond more adaptively to internal fluctuations during performance. While PST has demonstrated utility for skill acquisition, its emphasis on controlling internal experiences is increasingly recognized as insufficient in sports where micro-instability, intrusive thoughts, and evaluative pressure are unavoidable. By integrating structured skill progression and behavioral rehearsal from PST (e.g., structured skill progression and behavioral rehearsal) with ACT’s core processes—acceptance, defusion, present-moment awareness, self-as-context, values, and committed action—the ACPT-S provides a hybrid framework that addresses both the technical and experiential dimensions of shooting performance.

Results from the pilot implementation indicated that athletes experienced meaningful functional improvements, including enhanced emotional acceptance, reduced rumination, improved attentional selectivity, and strengthened value-oriented commitment. These outcomes align with the conceptual position that psychological flexibility serves as a central performance mechanism in contexts requiring sustained attentional steadiness and tolerance of internal variability. The shift reported by athletes—from experiential control strategies to acceptance- and values-based responding—suggests that the ACPT-S supports a deeper metacognitive engagement with performance-relevant experiences.

Expert evaluation further supported the conceptual validity and applied relevance of the program. All components exceeded the required Content Validity Ratio threshold, indicating strong professional consensus. Qualitative insights from experts emphasized the appropriateness of the embodied elements, the alignment between ACT principles and sport-specific routines, and the effectiveness of the sequential program structure. Importantly, the validation process employed in this study—integrating needs assessment, iterative refinement, pilot feedback, and expert review—represents a rigorous formative evaluation consistent with contemporary standards in intervention development research.

The theoretical contribution of this study lies in its integration of ACT’s functional contextual foundations with sport-specific attentional and perceptual-motor demands. Rather than treating PST and ACT as competing frameworks, the ACPT-S demonstrates how PST’s structured progression can be combined with ACT’s acceptance-based mechanisms to produce a comprehensive and ecologically valid performance intervention. This hybrid model offers a conceptual pathway for addressing longstanding gaps in the literature, particularly the limited attention to experiential variability, perfectionistic pressure, and internal fluctuation tolerance in precision sports such as shooting. The embodied implementation of ACT processes—such as breath–trigger synchronization, proprioceptive grounding, and micro-instability tolerance—further positions ACPT-S as an innovative model capable of bridging psychological processes with the psychophysiological microstructure of elite shooting.

In summary, this study provides an evidence-informed and contextually grounded framework for enhancing psychological flexibility in elite shooters. Although preliminary, the findings suggest that acceptance-based performance training offers a promising alternative to traditional cognitive-control approaches and may be particularly beneficial in sports where internal experiences cannot be reliably controlled. Future research should implement longitudinal and controlled designs to evaluate the causal effects of ACPT-S, explore its adaptability across various precision-based and endurance sports, and investigate the neural and attentional mechanisms that may underlie the observed psychological changes.

This program contributes to a growing body of work advocating acceptance-based performance models and provides a foundational step toward establishing psychological flexibility as a core competency in high-stakes athletic performance.

## 6. Limitations

Despite its contributions, several limitations should be acknowledged to ensure a balanced and rigorous interpretation of the findings.

First, the pilot phases involved relatively small samples, particularly Phase 1 (n = 4). Although such constraints are common in elite sport research—where access to high-performance athletes is inherently limited—the modest sample size restricts statistical generalizability. Within the logic of formative evaluation, these samples were appropriate for developmental refinement; however, future studies should employ larger and more heterogeneous samples across age groups, competitive levels, and cultural contexts to strengthen external validity.

Second, although ACPT-S was conceptually positioned as an advancement over conventional PST, the present study did not include a direct comparison group receiving PST. As a result, while theoretical and experiential limitations of PST (e.g., uniform routines, control-oriented strategies, limited acceptance training) were addressed, comparative effectiveness remains empirically untested. Future research should incorporate randomized controlled trials or non-inferiority designs directly contrasting ACPT-S with established PST protocols.

Third, the validation relied on formative evaluation indicators—needs assessment, iterative pilot implementation, and expert review—rather than long-term performance outcomes. This approach was appropriate for early-phase program development and ensured ecological validity; however, the absence of longitudinal follow-up limits conclusions about durability. Subsequent studies should evaluate ACPT-S across competitive seasons to assess sustained psychological and performance effects.

Fourth, the delivery format was fully online due to contextual restrictions (e.g., COVID-19). Although feasible and positively evaluated, athletes noted that certain experiential or metaphor-based exercises may benefit from in-person facilitation. Hybrid delivery models should therefore be explored to optimize both accessibility and experiential depth.

Finally, although metaphors and experiential tasks were culturally adapted for Korean elite shooters, interpretation of ACT metaphors can vary across linguistic and cultural contexts. Given that ACT emphasizes relational and linguistic processes, cross-national adaptation and cultural validation studies will be essential to ensure global applicability of ACPT-S.

By acknowledging these limitations, this study provides a transparent account of its methodological scope and identifies clear directions for rigorous, comparative, and longitudinal research moving forward.

## 7. Conclusions and Recommendations

This study developed and preliminarily validated an ACT-based psychological skills training program (ACPT-S: Acceptance and Commitment Performance Training for Elite Shooters) tailored to the psychophysiological demands of elite shooting. The needs assessment identified core psychological challenges—including worry, attentional instability, and emotional rigidity—that directly informed program construction. Through iterative pilot testing and expert validation, ACPT-S demonstrated feasibility, contextual fit, and conceptual clarity, establishing a robust foundation for subsequent empirical evaluation.

The study offers several theoretical and practical contributions. First, it introduces an integrative framework that bridges ACT’s six core processes with structured progression principles derived from traditional PST. This hybridization addresses long-standing limitations of PST—particularly its emphasis on control-oriented strategies—while preserving its strengths in systematic skill development. In doing so, ACPT-S positions psychological flexibility as a central performance competency rather than a clinically oriented construct, expanding ACT’s applicability beyond therapeutic settings into high-performance sport.

Second, the finalized ACPT-S model operationalizes ACT processes into seven performance-relevant modules: self-understanding, value clarification, acceptance, cognitive defusion, present-moment awareness, self-as-context, and committed action. These modules were culturally and contextually adapted for elite shooters, ensuring that the training content aligns with the microstructure of precision performance. Importantly, this study reconceptualizes ACT as a proactive training paradigm that enhances resilience, attentional regulation, and meaning-oriented engagement—outcomes that are essential for sustained excellence in elite sport.

Third, ACPT-S provides a structured alternative to conventional PST by embedding acceptance-based mechanisms into sport-specific routines. This approach promotes adaptive functioning under pressure, helping athletes maintain openness, clarity, and value-driven behavior in high-stakes environments. The model’s flexibility also suggests potential adaptability across different sports and competitive levels, highlighting its broader utility within performance psychology.

Future research (Study 2) should evaluate the long-term impact of ACPT-S on competitive outcomes, its comparative efficacy against established PST programs, and its applicability across diverse sport contexts. Incorporating digital technologies—such as biofeedback, attentional tracking sensors, or app-supported self-practice modules—may enhance scalability and provide more precise assessments of psychological change over time.

In conclusion, this study reframes ACT not as a clinical intervention but as a rigorously validated, performance-oriented training framework. By embedding acceptance-based processes within the architecture of elite sport training, ACPT-S contributes a novel, evidence-informed paradigm that advances psychological resilience through flexibility, enhances attentional precision through present-moment engagement, and promotes sustainable excellence through values-driven action. It thereby establishes a foundation for the next generation of mental performance training in high-performance environments.

## Figures and Tables

**Table 1:** Design of the Preliminary Study.

Group (R)	Pre-Test	Intervention	Post-Test
R_1_	Q_1_	X_1_	Q_2_

X = ACPT-S intervention.

**Table 2:** Participant Characteristics of Pilot Study 1 (2021).

No	Gender	Type	Age	Years of Experience
1	M	Pistol	23	11
2	M	Rifle	23	8
3	F	Rifle	23	7
4	F	Rifle	23	10

*n* = 4, *M*(age) = 23.0 years; *M*(experience) = 9.0 years.

**Table 3:** Participant Characteristics of Pilot Study 2 (2023–2024).

No	Gender	Type	Age	Athletic Experience (Years)
1	M	Pistol	37	23
2	M	Pistol	33	18
3	M	Pistol	35	20
4	M	Pistol	23	8
5	F	Pistol	29	14
6	M	Pistol	32	16
7	M	Pistol	27	15
8	M	Rifle	25	20
9	M	Rifle	23	17
10	M	Rifle	30	12
11	M	Rifle	28	13
12	M	Rifle	32	16
13	F	Rifle	30	15
14	F	Rifle	29	14
15	F	Rifle	28	11

*n* = 15, *M*(age)= 29.4 years; *M*(experience)= 15.5 years.

**Table 4:** Characteristics of the Expert Panel on Sport and ACT Counseling (n = 10).

**Group**	**Gender**	**Coaching Experience (Years)**	**Athletic Experience (Years)**	**Position**		**Sport**
Shooting Expert	M	30	10	National Coach		Shooting
	F	-	10	Active Athlete		Shooting
**Group**	**Gender**	**Teaching** **Experience** **(years)**	**Research** **Experience** **(years)**	**Sport** **Psychology** **Counseling** **Experience** **(years)**	**ACT** **Counseling** **Experience** **(years)**	**Sport**
Sport Psychology Counseling Expert	F	25	20	20	6	Shooting
	M	24	20	18	18	-
	M	13	13	13	-	Golf
	M	20	15	10	1	Track
	M	12	14	-	13	-
	M	10	16	10	5	Taekwondo
	M	9	12	9	-	-
	M	3	8	7	-	-

ACT = Acceptance and Commitment Therapy. “-” indicates data not available or not applicable. Gender is coded as M (Male), F (Female).

**Table 5:** Content Categories Derived from Needs Assessment 1 (Conventional Qualitative Content Analysis).

What Psychological Aspects Do You Feel Need Improvement?		
Response Content	Category	Percentage (%)
·Frequently worried ·Useless thoughts ·Reduce rumination	Worries & Overthinking	21.4
·Feeling distracted due to excessive thoughts when anxious ·Need to avoid being anxious ·Extreme tension	Anxiety & Tension	21.4
·Low mental resilience ·Mental weakness ·Easily losing mental focus	Mental Toughness	21.4
·Concentration Issues	concentration	7.1
·Thinking without becoming emotionally reactive or stressed	Emotional Regulation	7.1
·Need to enhance self-confidence	Self-Confidence	7.1
·Expressing emotions ·Need to practice expressing desires	Others	14.3

**Table 6:** Content Categories Derived from Needs Assessment 2 (Conventional Qualitative Content Analysis).

What Kind of Support Would You Like to Receive from Psychological Skills Training?		
Response Content	Category	Percentage (%)
·Mental training ·Learning how to manage mental skills ·Mental strengthening ·Support to become a better athlete becoming more mentally resilient and consistent	Mental Toughness Enhancement	42.9
·How to manage tension ·Overcoming tension	Tension Management	14.3
·Learning how to cope with anxiety ·Getting out of anxious states	Anxiety Regulation	14.3
·Maintaining focus in distracting or stressful situations	Concentration	7.1
·Emotional regulation	Emotional Control	7.1
·Learning how to overcome slumps	Overcoming Slumps	7.1
·Not giving up easily	Persistence & Motivation	7.1

**Table 7:** Content Categories Derived from Process Evaluation (Conventional Qualitative Content Analysis).

Participants’ Reflections on the Benefits of Acceptance and Commitment Performance Training	
A	Observing my thoughts and myself from a third-person perspective without judgment helped me realize that the problems were not as severe as they seemed. Applying this perspective to my daily life was highly effective—it helped relieve more than half of the worries and concerns that had been overwhelming my mind.
B	I enjoyed the activity where we visualized the most joyful moments in our lives. It was fun and engaging.
C	The training that involved defining elements of ACT in my own words was especially meaningful. Through this process, I came to trust myself more and realized that the answers lie within me. I also learned to better understand my thoughts and emotions and to view myself more objectively.
D	Writing a letter of encouragement to myself and setting personal exercise goals were particularly helpful. Because the sessions involved only a small number of participants, each individual could fully engage in the training and accurately identify areas for personal improvement.

**Table 8:** Content Categories Derived from Outcome Evaluation (Conventional Qualitative Content Analysis).

Participants’ Reflections on Psychological Changes after ACT-Based Training	
A	During the course, I applied what I learned to address major worries and concerns and found it very effective. Negative thoughts became more positive, and by looking at my problems from a third-person perspective, I was able to approach them more calmly.
B	I now focus only on the problems I can solve and no longer dwell on those I cannot.
C	I used to suppress or ruminate on negative emotions and thoughts. Through the training, I learned to face and accept them from a third-person perspective. Now I can respond more adaptively to similar situations.
D	I gained insight into what is needed across past, present, and future timelines.

**Table 9:** Evaluation CVR Scores.

No	Contents of the Question	N	ne	CVR
1	Is the training suitable for field application?	10	9	0.80
2	Are the training objectives and activity content appropriate?	10	9	0.80
3	Is the training suitable for university and professional shooting athletes?	10	9	0.80

**Table 10:** Summary of Expert Panel Feedback by Training Element.

Category	Summary of Expert Opinions
Concrete Explanation of Concepts	It is necessary to provide appropriate instructional materials to support conceptual understanding of ACT and psychological skills. Training should clarify the purpose of each session so athletes understand why it is being conducted. Using concrete examples and explaining the meanings of key terms can improve athletes’ comprehension.
Adjustment of Training Duration	Allocating 20 min to explain core concepts may result in disengagement. Rather than including excessive content, training should be structured in a more concise and segmented manner to encourage athletes’ open engagement. Detailed preparation is necessary to effectively conduct multiple activities within limited time. Participants need sufficient time to understand, reflect, and complete worksheets—therefore, current time allocations may be too tight.
Revision and Supplementation of Program Content	Instead of setting long-term goals within a one-year frame, goals should be framed broadly in terms of the athlete’s identity to enhance psychological flexibility. For athletes who struggle with visual expression, written alternatives should be provided. Adding open-ended sections instead of fixed checklists allows athletes to express individual values and thoughts. Self-reflection should be prioritized, and session order may need to be rearranged accordingly. Limiting practice to within-session tasks may be burdensome; training should be designed to allow for ongoing practice and follow-up. Experts viewed the program as highly beneficial and emphasized that sustained application would further enhance its effectiveness.

## Data Availability

The raw data supporting the conclusions of this article will be made available by the authors upon reasonable request.

## Abbreviations

The following abbreviations are used in this manuscript:

ACT	Acceptance and Commitment Therapy
ACPT-S	Acceptance and Commitment Performance Training for Elite Shooters
PST	Psychological Skills Training
